# Comparative Quantitative Study on Phytochemical Contents and Antioxidant Activities of* Capsicum annuum* L. and* Capsicum frutescens* L.

**DOI:** 10.1155/2019/4705140

**Published:** 2019-04-11

**Authors:** Tomi Lois Olatunji, Anthony Jide Afolayan

**Affiliations:** Medicinal Plants and Economic Development (MPED) Research Centre, Department of Botany, University of Fort Hare, Alice, 5700, South Africa

## Abstract

The variabilities in the phytochemical contents and antioxidant activities of four varieties of two closely related cultivated* Capsicum* species,* C. annuum* and* C. frutescens*, were examined as an additional tool for establishing their phylogenetic relatedness and for breeding purposes. The methodology involved the use of ethanolic and aqueous extractions for the determination of the phytochemical and antioxidant properties. The phytochemical contents including total flavonoid, total phenol, and proanthocyanidins were evaluated spectrophotometrically while the antioxidant activities were determined by 2,2-diphenyl-1-picrylhydrazyl (DPPH), 2,2′-azino-bis(3-ethylbenzothiazoline)-6-sulfonic acid (ABTS), nitric oxide (NO), and phosphomolybdenum assays. To point out the relationship among the varieties, a dendrogram based on the antioxidative phytochemical contents was constructed using the unweighted pair group method with arithmetic mean (UPMGA) cluster analysis. In all, aqueous extracts gave higher yield while ethanolic extracts showed higher phytochemical content across the varieties. Significant variations were observed among the varieties in relation to their phytochemical constituents and antioxidant activities. Dendrogram obtained from multivariate analysis distinguished the two* Capsicum *species. The first cluster contained only* C. frutescens var. baccatum* while the second cluster contained the three varieties of* C*.* annuum* species in subclusters, signifying the close genetic affinity among the three varieties. It also revealed that the four varieties are of a common progenitor. Information from this study gives additional evidence of chemotaxonomic significance and baseline data for effective selection of suitable parental genotypes in breeding for nutritional and pharmacological purposes.

## 1. Introduction

Plants with several bioactive compounds are increasingly gaining attention because of their effectiveness in improving human health and nutrition [1–3]. The correct identification of these plant species with medicinal importance is the first and basic step in any improvement programme. This allows reliable and effective selection of suitable parental genotypes (with authentic purity and quality) in plant breeding programmes that are developed for various nutritional and pharmacological purposes [4–6].

The genus* Capsicum* with several universal English names, which include hot pepper, chile pepper, chili, sweet pepper, and bell pepper, belongs to the* Solanaceae* family [7].* Capsicum* consists of about 27 species with five of these being widely cultivated. These are* C. annuum *L*., C. chinensis* Jacq.*, C. frutescens *L*., C. pubescens *R., and* C. baccatum *L. [7].* Capsicum *spp. are important and popular vegetables and spices that are cultivated in the tropical and subtropical regions of the world [8]. They are vastly valued not only because of their economic importance but also for their rich nutritional value. The fruit of pepper contains a range of bioactive phytochemicals including flavonoids, carotenoids, phenolics, and other antioxidant compounds [9]. Besides the nutritional benefits of pepper and their use as food additives, the hot* Capsicum* spp. (due to their capsaicin content) have a significant role in pharmacy and are currently used for different therapeutic purposes. The active principle in red peppers responsible for their medical and pharmacological use is the pungent alkaloid called capsaicinoids [10, 11].

Several classes of phytochemicals (phenolic compounds, particularly flavonoids and phenolic acid) and antioxidants are sufficiently available in high amounts in vegetables and fruits; thus they form an important part of human diet [1–3, 12]. They are known to protect the body cells by fighting off free radicals in the body by disallowing oxidation process. Series of reactions initiated by free radicals cause damage to membrane and disruption of metabolic pathways thereby increasing mutations in DNA and alteration of platelet function among others [1–3, 12]. Since numerous studies have suggested that eating foods rich in phytochemicals and antioxidants has been linked with lessened risk of certain forms of cancer, stroke, and cardiovascular diseases, much attention is recently given to natural foods especially vegetables rich in these compounds [13, 14]. Several studies have documented the effectiveness of the antioxidative components of various pepper species [9, 11, 15]. For example, Loizzo* et al*. [16] reported the inhibitory effect of* C. annuum* var.* acuminatum* on the enzyme acetylcholinesterase (AChE). The inhibition of this enzyme is one of the therapeutic methods for the symptomatic management of Alzheimer's disease. The appreciable high amount of phenols and flavonoids in the ethanolic extracts of* C. annuum* var.* acuminatum* small and* C. annuum* var.* cerasiferum* contributed to their antioxidant capacities where the stable free radical DPPH was reduced to yellow colored DPPH [17]. Similarly, Takahashi* et al*. [18] reported high antioxidant properties of the fruit extracts of* C. frutescens* from green to red stages based on the oxygen radical absorbance capacity (ORAC) and DPPH tests. In another study, methanolic extracts from* C. annuum* L. were reported to inhibit 4-hydroxy-2nonenal-induced and H_2_O_2_-induced DNA damage; this study was done on human leucocytes and a potential toxicity was reported against HT-29 cells [19]. Thus, peppers are among the vegetables that provide a rich source of various bioactive compounds with potential health-improving properties. However, the occurrence and distribution of the various types of phytochemicals are under genetic control; they differ by genotype and maturity phase in plants [20]. Phytochemical taxonomists have considered them as useful taxonomic markers. The variation in phytochemical constituents can be exploited in taxonomic studies and can be used to establish phylogenetic relatedness in plants.

**Figure 1:**
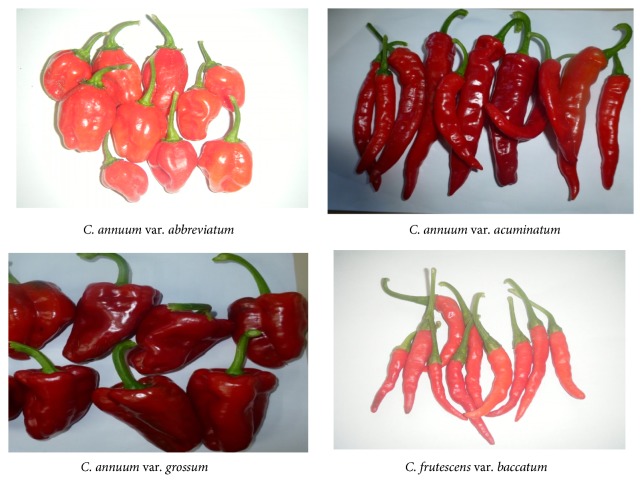
Images showing the typical shapes and sizes of the four varieties of the cultivated* Capsicum* species in West Africa assessed for their phytochemical content and antioxidant activities.

In West Africa, the genus* Capsicum* is represented by two cultivated species, namely,* Capsicum annuum* and* Capsicum frutescens*, with numerous varieties. Because the two species are morphologically and closely related, there has been considerable debate on their status as two separate species or just varieties of one species. Based on the sizes and morphological differences between and within the cultivated pepper, four varieties are recognized, namely,* Capsicum annuum* var.* abbreviatum*,* Capsicum annuum* var*. acuminatum*,* Capsicum annuum* var.* grossum*, and* Capsicum frutescens* var.* baccatum*, which are locally known by the Yoruba people as rodo, sombo, tatase, and wewe, respectively (Figure 1). Comparative profiling of bioactive compounds can complement genomic and taxonomic identification of plant species [21]. There is dearth of information on the characterization of bioactive compounds of the cultivated* Capsicum* spp. As the search for clues to understand the genetic relationship among the cultivated* Capsicum* spp. increases, this study is geared at evaluating and comparing the bioactive compounds of the varieties of the cultivated* Capsicum* species and determining if they can be used as an additional tool for establishing phylogenetic relatedness in these species and, also, to identify varietal groups with close relationship that can be useful for selecting potential parents in breeding program.

## 2. Materials and Methods

### 2.1. Collection and Growth of Plant Materials

Mature fruits of the four cultivated varieties of* Capsicum annuum* and* Capsicum frutescens* were obtained from markets (Oja-tuntun in Ilorin, Kwara, Babban Kasuwan in Gombe, Kontagora in Niger, Ogbete in Enugu, Oja-Oba in Ado-Ekiti, and Ebele market in Edo) in major geographical zones in Nigeria. Seeds in the fruits were first removed, sun dried (for 3 days), and stored (for 2 weeks) at room temperature of about 15-25°C in paper bags and were later used for planting. Planting was done in plastic pots in the Green house of the University of Fort Hare between September 2017 and February 2018. The voucher specimens of all experimental varieties were deposited at the University of Ilorin's herbarium, with the voucher numbers UIH 001/532, UIH 002/532, UIH 003/532, and UIH 004/751 for* C. annuum* var.* abbreviatum*,* C. annuum* var.* acuminatum*,* C. annuum* var.* grossum*, and* C. frutescens* var.* baccatum*, respectively. Mature fruits of the four varieties of the* Capsicum* spp. were harvested and quantitatively analyzed for total phenols, flavonoids, and proanthocyanidins contents. The antioxidant activities examined include DPPH, ABTS, NO, and phosphomolybdenum assays.

### 2.2. Reagents and Chemicals

All the reagents and solvents used were of analytical grade and were purchased from Merck and Sigma-Aldrich, Gauteng, South Africa. These include glacial acetic acid(CH_3_COOH), potassium acetate (CH_3_CO_2_K), anhydrous sodium carbonate (Na_2_CO_3_), aluminum trichloride (AlCl_3_), Folin-Ciocalteu reagent, 2,2 diphenyl-1-picrylhydrazyl (DPPH), 2,2′-azino-bis (3-ethylbenzthiazoline-6-sulfonic acid) (ABTS), sodium nitrite (NaNO_2_), sodium chloride (NaCl), vanillin, ferric chloride (FeCl_2_), butylated hydroxyl toluene (BHT), ascorbic acid, rutin, ethanol, hydrochloric acid, sodium hydroxide, phosphate buffer, potassium ferricyanide [K_3_Fe(CN)_6_], ammonium molybdate, sodium phosphate, trichloroacetic acid (TCA), and sodium nitroprusside (Na_2_[Fe(CN)_5_NO]_2_H_2_O).

### 2.3. Preparation of Extracts

Mature fruits of the four varieties of the* Capsicum* spp. were washed with distilled water and blotted gently using a paper towel to remove excess water. The fruits were separately placed under constant flow of air until fully dried and then pulverized in an industrial electric blender (Polymix PX-MFC 90D Switzerland), sealed in labeled plastic bags, and stored at 4°C in the refrigerator until extraction, using water and ethanol as the solvents. Extraction was done by weighing 65g of each ground sample into separate labeled conical flasks containing 600mL of the solvents and shaken for 48 hours on a mechanical shaker (Gallenkamp Orbital Shaker). The crude extracts were filtered under pressure using a Buchner funnel, vacuum pump, and Whatman No. 1 filter paper. The aqueous filtrate of each sample was chilled at -40°C with a chiller (PolyScienceAD15R-40-A12E, USA) and concentrated using a freeze-dryer (Vir Tis benchtop K, Vir Tis co, Gardiner, NY.) for 48 hours. Similarly, the ethanolic extracts were concentrated to remove the solvents using a rotary evaporator (Strike-202 Steroglass, Italy). Percentage yield of aqueous and ethanolic extracts of each sample were determined and recorded. The extracts were stored at 4°C until further analysis.

### 2.4. Quantitative Phytochemical Evaluation

#### 2.4.1. Total Phenol Content

Total phenols in each plant extract were assayed spectrophotometrically using the Folin–Ciocalteu's reagent method as described by [22] with slight modifications. Briefly, 0.5mL of each plant extract (1mg/mL) and gallic acid (0.02 to 0.1mg/mL) were pipetted into separately labeled test tubes. 2.5mL of 10% (v/v) Folin–Ciocalteu's reagent was added and the mixture vortexed. To the solution, 2mL of 7.5% w/v Na_2_CO_3_ was added, vortexed, and incubated at 40°C for 30 mins. After incubation, the absorbance at 765nm was read using Hewlett Packard VR-2000 spectrophotometer alongside methanol as blank. All samples were analyzed in triplicate. The total phenol in each variety was expressed as milligram per gram of extract's total phenol content in gallic acid equivalent (mg GAE/g) using the standard curve: y = 0.2281x-0.0264, R^2^ = 0.964, where R is the determined coefficient, x is the concentration, and y is the absorbance.

#### 2.4.2. Total Flavonoid Content

Total flavonoids in each variety were determined using the colorimetric aluminium chloride assay as described by [2]. Briefly, distilled water (2mL) and 0.15mL of 5% NaNO_3_ were mixed and added to an aliquot of each extract and were allowed to stand for 5 minutes. Thereafter, 0.15mL of 10% AlCl_3_ was added to the solution. After 5 minutes, 1mL of 4% NaOH was added. The solution was vortexed and incubated at 40°C for 15 mins. Varying concentrations of the standard solution (quercetin) were also prepared following the same procedure. Absorbance of the solution was measured at 510 nm and total flavonoid contents in each variety were expressed as mg/g quercetin equivalents (mg QE/g) of extract: y = 0.3658x+ 0.0356, R^2^= 0.9618. All samples were analyzed in triplicate.

#### 2.4.3. Proanthocyanidins (Total Condensed Tannins)

Total proanthocyanidins in each sample were determined using the method of [2]. Briefly, to 0.5mL of each test sample, 3mL of 4% vanillin-methanol solution and 1.5mL of HCl were added. The mixture was vortexed and incubated at 27°C for 15 minutes. Absorbance was measured and read at 500nm. The amount of total condensed tannin was expressed as mg/g dry weight of catechin equivalent (mg CE/g) of the extract; y = 4.751x- 0.4801, R^2^= 0.9437. All samples were analyzed in triplicate.

### 2.5. Evaluation of Antioxidant Activity

The antioxidant activities of the four varieties of the cultivated* Capsicum* species were determined using DPPH, ABTS, nitric oxide, and phosphomolybdenum (total antioxidant capacity) assays.

### 2.6. DPPH (2, 2-Diphenyl-1-Picrylhydrazyl) Radical Scavenging Activity Assay

DPPH radical scavenging activity for each variety was determined by a modified method previously described by [2]. Briefly, a reaction mixture that contained 2.5mL of DPPH solution (0.13mM) and 2.5mL of each plant extract or standard (rutin) dissolved in methanol at varying concentrations of 0.005- 0.08mg/mL was thoroughly vortexed and kept in the dark for 30 mins. The absorbance of the mixture was measured spectrophotometrically at 517nm against the blank and control. The DPPH radical scavenging activity was calculated according to Iqbal* et al*. [23] Consider the following equation:

(1) %  DPPH  scavenging  activity=Abs  DPPH−Abs  SampleAbs  DPPH)×100

The relationship between percentage inhibition and equivalent sample concentration was plotted to determine the half-inhibitory concentration (IC_50_) value of each sample.

### 2.7. ABTS (2, 2′-Azino-Bis (3-Ethylbenzothiazoline)-6-Sulfonic acid) Radical Scavenging Activity

A modified method of [24] was adopted for the determination of ABTS activity of each plant extract. Equal proportions (1:1) of ABTS (7mM) mixed with K_2_S_2_O_8_ (2.45mM) were left in the dark for 18h for the formation of a green colored ABTS^+^. ABTS^+^ solution was further diluted with methanol (1:50v/v) to an absorbance of 0.700±0.005 at 734nm which served as the working solution. Then, 1mL of plant extracts or standard drugs (rutin, gallic, and BHT) at varying concentrations (0.005-0.08mg/mL) was mixed with the resulting ABTS^+^ solution and was allowed to stand in the dark for 7 minutes. Thereafter, the absorbance at 734nm was read against methanol (blank). The percentage inhibition of samples and standards was calculated using Khatua's* et al*. [25] Consider the following equation:

(2) %  Inhibition=Absorbance  of  control−Absorbance  of  sampleAbsorbance  of  control×100.

The sample concentrations providing 50% (IC_50_) of antioxidant activity were calculated from graph by plotting percentage inhibition of ABTS^+^ by the samples against the corresponding sample's concentration.

### 2.8. NO (Nitric Oxide) Scavenging Activity

Sample's inhibitory capabilities against NO radicals were determined using a modified method of [2]. Briefly, 10mM phosphate buffer saline (pH 7.4) was dissolved in 10mM sodium nitroprusside. Two milliliters (2mL) of this solution were added to 0.5mL of plant extract and standard drugs (rutin and BHT) at varying concentrations (0.025-0.4mg/mL). After an incubation period of 2.5h at 27°C, 0.5mL of Griess reagent {1mL sulphanilamide (0.33% dissolved in 20% glacial acetic acid) with 1mL 0.1% w/v 1-naphthylethylenediamine} was added to the mixture and incubated at room temperature for 30 mins. The absorbance at 540 nm was read against the blank (methanol) and amount of nitric oxide radicals inhibited by the extract was determined using the equation:

(3) %  NO  Scavenging=Abs  control−Abs  sampleAbs  control×100

The sample concentrations providing 50% (IC_50_) of antioxidant activity were calculated from graph by plotting percentage scavenging of NO of the samples against the corresponding sample's concentration.

### 2.9. Phosphomolybdenum Assay {Total Antioxidant Capacity (TAC)}

The total antioxidant capacity (TAC) of the plant extract was determined following a modified protocol of [2]. Briefly, 3mL of reagent solution (containing 28mM sodium phosphate, 0.6 M sulfuric acid, and 4mM ammonium molybdate) was added to 0.3mL of plant extracts and different standard drugs (rutin, BHT, and gallic acid) at different concentrations of 0.025-0.400mg/mL. Following an incubation period of 90 mins at 90°C in a water bath and after cooling to room temperature, the absorbance at 695 nm was measured against the blank (methanol) using a spectrophotometer. The percentage inhibition was calculated as

(4) %  TAC  inhibition=Abs  sample−Abs  controlAbs  sample×100.

The sample concentrations providing 50% (IC_50_) inhibitory effect were calculated from graph by plotting percentage inhibition of samples against the corresponding sample's concentration.

### 2.10. Statistical Analysis

The results are expressed as mean values of three replications ± standard deviation (SD). Statistical analysis was performed by analysis of variance (ANOVA). Where the data showed significance difference (p < 0.05) among the varieties, a mean separation was done using Fischer's LSD with the aid of MINITAB 17 statistical package. To point out the relationship among the varieties of the* Capsicum* spp. studied, a dendrogram was constructed using the unweighted pair group method of arithmetic averages (UPGMA) by using the paleontological statistics (PAST) software, version 2.

## 3. Results

The percentage ethanolic and aqueous yields of each variety after extraction are presented in Table 1. Across the varieties, the aqueous extracts gave higher percentage yield in comparison with the ethanolic extracts.

### 3.1. Phytochemicals

Phytochemical analysis showed that phenol, flavonoid, and proanthocyanidins were present in the extracts of the four varieties of the cultivated* Capsicum *species studied and the mean values of their phytochemical contents for both ethanolic and aqueous extracts are presented in Table 2. Overall, the varieties under study showed higher phytochemical constituents in ethanolic extracts compared to those of aqueous extracts (Table 2). The TP content for ethanolic extracts ranged from 200.69 ±11.53mg GAE/g DW in* C*.* annuum* var.* acuminatum* to 272.47± 7.38mg GAE/g DW in* C*.* annuum* var.* grossum*. TP content in* C*.* annuum* var.* grossum* was significantly higher than those of other three varieties (p< 0.05) (Table 1). Also, TP contents in* C*.* annuum* var.* abbreviatum* and* C*.* frutescens* var.* baccatum* showed no significant difference but were significantly higher than* C*.* annuum* var.* acuminatum*. Among the four varieties of pepper, TP content for aqueous extract was highest in* C*.* frutescens* var.* baccatum* showing 90.86±3.78 mg GAE/g DW and was lowest in* C*.* annuum* var.* grossum* showing 57.36± 2.50 mg GAE/g DW (Table 2).

As shown in Table 2, total flavonoid (TF) contents of both ethanolic and aqueous extracts differed among the pepper varieties but not all of them differed significantly. The highest and lowest TF contents of ethanolic extracts were obtained for* C. annuum var. grossum *(1630.53± 86.96mg QE/g DW) and* C. frutescens var. baccatum* (867.241± 53.87mg QE/g DW), respectively. However,* C. annuum var. grossum* showed no significant difference with* C. annuum var. abbreviatum* in their TF contents of ethanolic extracts but were significantly different from* C. annuum var. acuminatum* and* C. frutescens var. baccatum *(Table 2). In the aqueous extracts, highest flavonoid content was recorded for* C. frutescens var. baccatum *while the least was recorded for* C. annuum var. grossum* (Table 2).

Proanthocyanidins (total condensed tannin TCT) contents showed no significant difference (p>0.05) in all the ethanolic extracts of all pepper varieties except in* C. annuum var. grossum. *The highest ethanolic extract of TCT content was recorded for* C. annuum var. grossum* (709.99± 5.50mg CE/g DW) while* C. frutescens var. baccatum *gave the lowest TCT contents (616.81±12.34mg CE/g DW). Similarly, TCT contents in all the aqueous extracts of the four pepper varieties showed no significant difference (Table 2).

### 3.2. Antioxidant Activity

#### 3.2.1. DPPH (2, 2-Diphenyl-1-Picrylhydrazyl) Radical Scavenging Activity Assay

**Figure 2:**
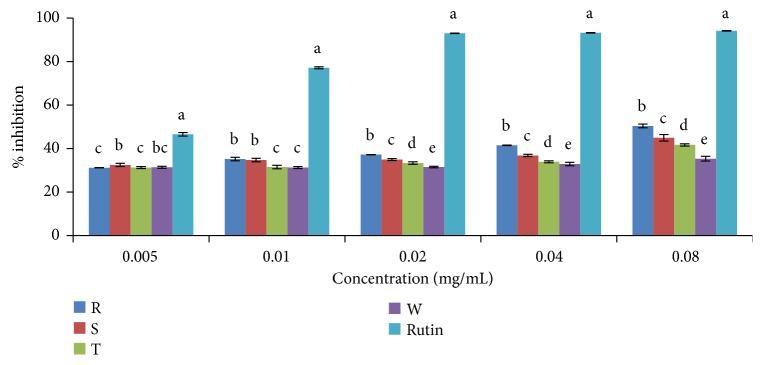
DPPH scavenging activity of ethanolic extract of the four varieties of* Capsicum *species. Values are mean ± SD of three replications. Set of bars having different letters are significantly different at (p<0.05). R=* C. annuum* var.* abbreviatum*, S=* C. annuum* var.* acuminatum*, T=* C. annuum* var.* grossum*, and W=* C. frutescens* var*. baccatum*.

**Figure 3:**
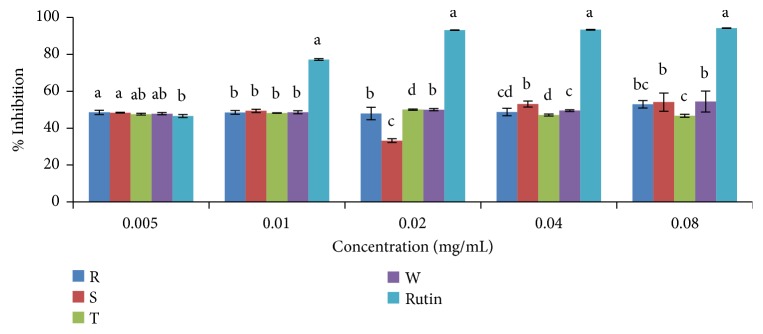
DPPH scavenging activity of aqueous extract of the four varieties of* Capsicum *species. Values are mean ± SD of three replications. Set of bars having different letters are significantly different at (p<0.05). R=* C. annuum* var.* abbreviatum*, S=* C. annuum* var.* acuminatum*, T=* C. annuum* var.* grossum*, and W=* C. frutescens* var*. baccatum*.

DPPH scavenging activities of ethanolic and aqueous extracts of the four pepper varieties in comparison to standard antioxidant (rutin) are shown in Figures 2 and 3, respectively. Scavenging activity of both ethanolic and aqueous extracts of the pepper varieties including the standard (rutin) was concentration dependent (Figures 2 and 3). The IC_50_ values (concentration of extracts/standard drug required to scavenge 50% of the radicals) are presented in Table 3, and this value was found to be inversely proportional to its scavenging activity. The standard drug (rutin) showed a stronger activity with an IC_50_ of 0.0056 mg/mL when compared to the plant extracts. In comparison to the ethanolic extracts, the aqueous extracts of the four pepper varieties gave higher scavenging activity. Accordingly, in the ethanolic extracts, DPPH radical scavenging activities based on IC_50_ values were in the order rutin >* C. annuum* var.* abbreviatum *> (*C. annuum* var.* acuminatum*,* C. annuum* var.* grossum*, and* C. frutescens* var.* baccatum*). The order of DPPH radical scavenging activity in the aqueous extract based on IC_50_ values was rutin >* C. annuum* var.* acuminatum* >* C. frutescens* var.* baccatum* >* C. annuum* var.* grossum* >* C. annuum* var.* abbreviatum*. (Figure 3; Table 3).

#### 3.2.2. ABTS (2, 2′-Azino-Bis (3-Ethylbenzothiazoline)-6-Sulfonic Acid) Radical Scavenging Activity

**Figure 4:**
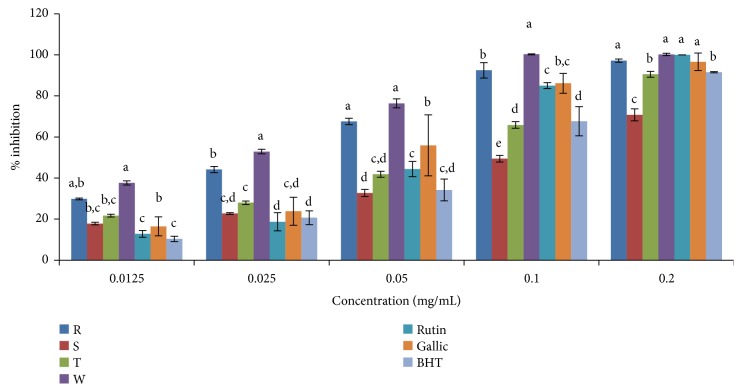
ABTS^+^ radical scavenging activity of ethanolic extract of the four varieties of* Capsicum *species. Values are mean ± SD of three replications. Set of bars having different letters are significantly different at (p<0.05). R=* C. annuum* var.* abbreviatum*, S=* C. annuum* var.* acuminatum*, T=* C. annuum* var.* grossum*, and W=* C. frutescens* var*. baccatum*.

**Figure 5:**
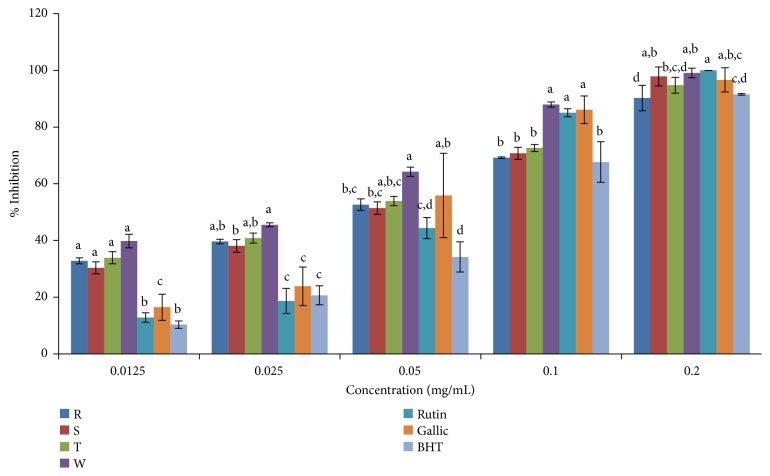
ABTS^+^ radical scavenging activity of aqueous extract of the four varieties of* Capsicum *species. Values are mean ± SD of three replications. Set of bars having different letters are significantly different at (p<0.05). R=* C. annuum* var.* abbreviatum*, S=* C. annuum* var.* acuminatum*, T=* C. annuum* var.* grossum*, and W=* C. frutescens* var*. baccatum*.

Across the four varieties of the cultivated* Capsicum* spp. studied, significant amount of ABTS^+^ scavenging activity was shown in both aqueous and ethanolic extracts and the scavenging activity was concentration dependent (Table 3, Figures 4 and 5). The ethanolic and aqueous extracts of* C. frutescens* var.* baccatum *scavenged this radical better than the standard drugs (rutin, gallic, and BHT) and other sample extracts. This is evident by its low IC_50_ values (Table 3).* C. annuum* var.* acuminatum* showed the lowest ABTS^+^ scavenging activity in both aqueous and ethanolic extracts when compared to other sample extracts. Overall, the ABTS^+^ antioxidant activities based on the IC_50_ values were in the order* C. frutescens* var.* baccatum *>* C. annuum* var.* abbreviatum* > gallic > rutin >* C. annuum* var.* grossum* > BHT >* C. annuum* var.* acuminatum* in ethanolic extracts and* C. frutescens* var.* baccatum *>* C. annuum* var.* grossum* > (*C. annuum* var.* abbreviatum*, gallic) >* C. annuum* var.* acuminatum* > rutin > BHT in aqueous extracts.

### 3.3. NO (Nitric Oxide) Scavenging Activity

**Figure 6:**
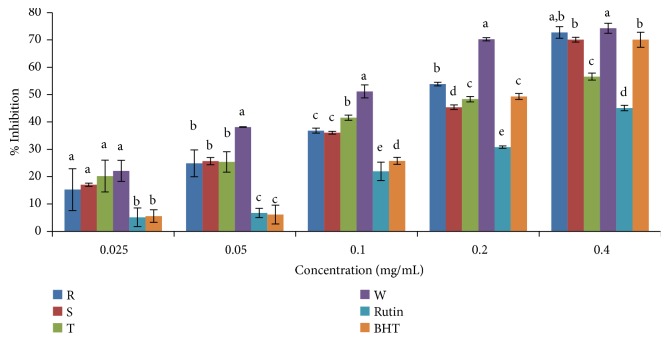
NO scavenging activity of ethanolic extract of the four varieties of* Capsicum *species.. Values are mean ± SD of three replications. Set of bars having different letters are significantly different at (p<0.05). R=* C. annuum* var.* abbreviatum*, S=* C. annuum* var.* acuminatum*, T=* C. annuum* var.* grossum*, and W=* C. frutescens* var*. baccatum.*

**Figure 7:**
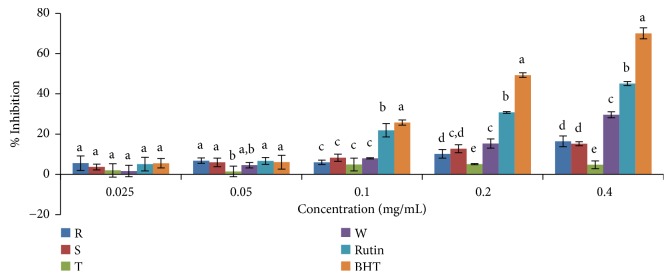
NO scavenging activity of aqueous extract of the four varieties of* Capsicum *species. Values are mean ± SD of three replications. Set of bars having different letters are significantly different at (p<0.05). R=* C. annuum* var.* abbreviatum*, S=* C. annuum* var.* acuminatum*, T=* C. annuum* var.* grossum*, and W=* C. frutescens* var*. baccatum*.

The antioxidant activity measured by NO was evident only in the ethanolic extracts of the varieties of* Capsicum* spp. studied. The % scavenging activity significantly differs (P< 0.05) among the four varieties of the cultivated* Capsicum* spp. in the ethanolic extract only at the concentration of 0.20 mg/mL and it ranged from 45.37% for* C. annuum* var.* acuminatum* to 70.24% for* C. frutescens* var*. baccatum*. The scavenging activity was found to increase with increasing concentration (Figure 6).* C. frutescens* var*. baccatum* showed NO scavenging activity higher than the standard drugs (rutin and BHT) and other plant extracts with an IC_50_ value of 0.0971mg/mL (Table 3). In general, based on the IC_50_ values, inhibitory capabilities against NO radicals in the ethanolic extracts were in the order* C. frutescens* var.* baccatum *>* C. annuum* var.* abbreviatum* > BHT >* C. annuum* var.* acuminatum* >* C. annuum* var.* grossum* > rutin. As shown in Figure 7, the aqueous extract of all samples showed very low NO scavenging abilities with IC_50 _values greater than the highest concentration used in this study (0.4 mg/mL).

### 3.4. Phosphomolybdenum Assay {Total Antioxidant Capacity (TAC)}

**Figure 8:**
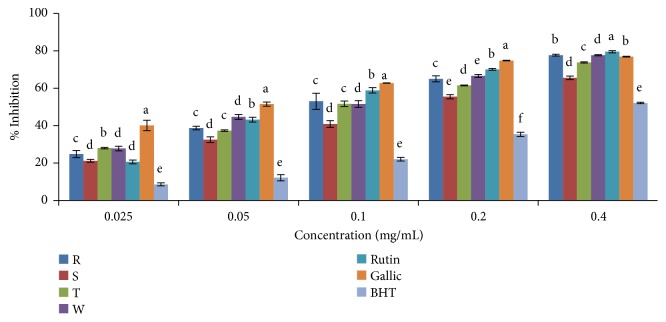
Total antioxidant capacity of ethanolic extract of the four varieties of* Capsicum *species. Values are mean ± SD of three replications. Set of bars having different letters are significantly different at (p<0.05). R=* C. annuum* var.* abbreviatum*, S=* C. annuum* var.* acuminatum*, T=* C. annuum* var.* grossum*, and W=* C. frutescens* var*. baccatum*.

**Figure 9:**
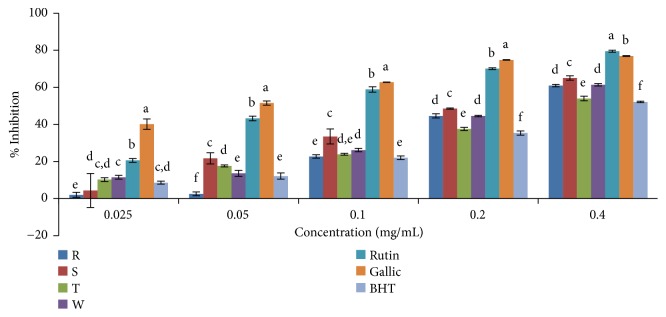
Total antioxidant capacity of aqueous extract of the four varieties of* Capsicum *species. Values are mean ± SD of three replications. Set of bars having different letters are significantly different at (p<0.05). R=* C. annuum* var.* abbreviatum*, S=* C. annuum* var.* acuminatum*, T=* C. annuum* var.* grossum*, and W=* C. frutescens* var*. baccatum*.

In the phosphomolybdenum assay, the total antioxidant capacity of standard drugs and plant extracts increased with increasing concentration in both ethanolic and aqueous extracts (Figures 8 and 9). The antioxidant capacities varied among the varieties of* Capsicum* spp. as it ranged from 65.55% for* C. annuum* var.* acuminatum* to 77.66% for* C. annuum* var.* abbreviatum* at the highest concentration (0.4mg/mL) in the ethanolic extract. The highest antioxidant capacity was exhibited in* C. frutescens* var*. baccatum* with an IC_50_ value of 0.0892mg/mL while the lowest activity was found in* C. annuum* var.* acuminatum* with an IC_50_ value of 0.1636mg/mL in the ethanolic extract (Table 3). Overall, the TAC was in the order gallic > rutin >* C. frutescens* var*. baccatum* >* C. annuum* var.* abbreviatum *>* C. annuum* var.* grossum* >* C. annuum* var.* acuminatum* > BHT in the ethanolic extract and gallic > rutin >* C. annuum* var.* acuminatum *>* C. annuum* var.* abbreviatum* >* C. frutescens* var*. baccatum* >* C. annuum* var.* grossum* > BHT in the aqueous extract.

### 3.5. Multivariate Analysis

**Figure 10:**
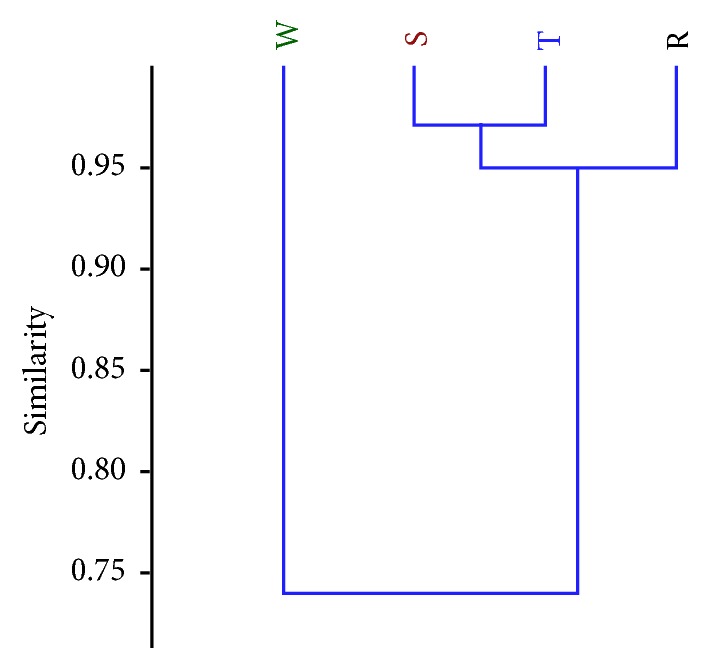
Dendrogram showing the relationship among the varieties of the cultivated* Capsicum* species based on their phytochemical and antioxidant contents. R=* C. annuum* var.* abbreviatum*, S=* C. annuum* var.* acuminatum*, T=* C. annuum* var.* grossum*, and W=* C. frutescens* var*. baccatum*.

Based on the multivariate analysis, the genetic similarity among the four varieties of pepper revealed that the four varieties are well related. The distance on the y-axis gives a measure of dissimilarity among the varieties and the value is largest where the varieties are most dissimilar (Figure 10). The dendrogram obtained from the analysis, defined by the phytochemical contents and antioxidant activities of the plants, revealed two major groups. The first group consisted of only* C. frutescens *var.* baccatum*, while the second group consisted of two subgroups,* Capsicum annuum* var.* acuminatum* and* C*.* annuum* var.* grossum*, indicating that these two are more closely related. Subgroup* Capsicum annuum* var.* acuminatum* consisted of two other clusters,* C*.* annuum* var.* abbreviatum* and* C*.* frutescens* var.* baccatum*, showing their relatedness to each other (Figure 10).

## 4. Discussion

Consumption of vegetables and fruits that are abundant in phytochemicals and antioxidants has been associated with lessened risks of various chronic disorders including cancer and cardiovascular diseases [26]. The production of phytochemicals with bioactive antioxidative properties by vegetables and fruits is affected by many factors particularly the genotype, thus making levels vary across varieties of same species of a given fruit or vegetable. Profiling of the bioactive constituents of different plant cultivars has been adopted for taxonomic purposes [20, 26]. Over the years, morphological and anatomical classifications have been used for taxonomic delineation and these approaches are considered traditional, but chemotaxonomic classification which involves profiling of chemical constituents is considered modern in the classification of plants [27]. Polyphenols, which are a major determinant of the antioxidant capabilities of plants, are among the important and largely exploited groups of compounds that have been utilized for chemotaxonomic classification. These compounds are of unrestricted occurrence in plants as they exhibit wide variation in distribution, quantity, and function, hence the justification for their use as a tool for delineation [26].

In this study, the variability in the phytochemicals and antioxidants contents of the four varieties of the cultivated* Capsicum *species in West Africa not fully investigated previously was used as an additional tool for their taxonomic delineation.

Generally, in this study, water produced the highest extraction yield across the four varieties of the* Capsicum* species in comparison to the ethanolic extracts yield. This yield of extraction is well correlated with the solvent character with the more polar solvent extracting higher than the intermediate or less polar solvent [28]. Such high extraction yield from aqueous extract has been reported by other researchers [2, 28].

Phenols, one of the major phytochemical constituents in a wide range of plants, have attracted the interest of several researchers since they show high level of antioxidant activity linked to the prevention of certain diseases in the human body such as cancer [24, 29]. This antioxidative activity could be attributed to their redox properties which increase their ability to adsorb and scavenge free radicals [24, 30]. In our study, the total phenolic contents were determined, and the result revealed high levels particularly in the ethanolic extracts. However, a large variability was seen across the varieties. In the ethanolic extract,* Capsicum annuum* var.* grossum* showed a significantly higher phenolic content (272.47± 7.38 mg GAE/g) than others while* C. frutescens var. baccatum* gave the highest total phenolic content (90.86± mg GAE/g) in the aqueous extract. The difference in the values of the phenolic contents from the two solvents used is an indication that they have different extractive capabilities for phenols from the study plants sampled. Similar observation was made by [31] where higher phytochemical content was recorded in the ethanolic extracts of some Nigerian spices including pepper when compared to their corresponding aqueous extracts. Zhuang* et al*. [29] reported variability in the phenolic contents of 9* Capsicum* varieties. Our result showed a higher phenolic content than those reported by studies on different sweet and hot peppers. This difference may be attributed to different cultivars used as well as the growing conditions [9, 29, 32].

Flavonoids account for 60% of total phenolics, making them the largest of naturally occurring phenolics. Their free radical scavenging capacity is attributed to their biological activity which includes antioxidant, anticancer, and anti-inflammatory [24, 27, 30, 33]. Ethanolic extracts of the four varieties of* Capsicum* species used in this study revealed high content of flavonoids in comparison to the aqueous extracts, with considerable variation in the flavonoid content of the four varieties. The flavonoid contents of the three varieties of* C*.* annuum* in both extracts were comparable to each other than the flavonoid content in* C*.* frutescens* var.* baccatum*. This result agrees with [34] who emphasized the role of plant genotype (species and varieties within a species) in the determination of bioactive contents as very close species or varieties in a species are likely to show similar values than species that are distantly related.

Proanthocyanidins also called condensed tannin are a group of polyphenolic bioflavonoids that have shielding effect in removing hydroxyl radicals [24]. They are unrestricted in occurrence in most plants and because of their antioxidant ability they are currently relevant in medicine and nutrition [35]. In this study,* Capsicum annuum* var.* grossum* contained significantly higher proanthocyanidin contents than other varieties in the ethanolic extract while the proanthocyanidin content was comparable across the four varieties in the aqueous extract. This chemical similarity in terms of their proanthocyanidin content is an indication of close relationship among the varieties [34, 36].

The evaluation of antioxidant activity gives valuable information about the functional quality of plant and can be used to better characterize plant species [37]. Because of the complexity in the nature of antioxidative compounds, the overall antioxidant capacities of vegetable crops cannot be determined based on a single assay; two or more assays have been suggested by several researchers to better evaluate the antioxidant capacities of plants [3, 37, 38]. In this study, we evaluated the antioxidant capacity of the varieties of* Capsicum* species using DPPH, ABTS, NO, and phosphomolybdenum assays.

Free radical scavenging is one of the known means through which antioxidants repress lipid oxidation that is resultant from free radicals. The steady free radical DPPH has been generally used to test the scavenging capacity of different plants that are rich in antioxidant [38, 39]. There has been current interest in the use of ABTS radical assay in evaluating the radical scavenging capabilities of several plants extracts because of the different report on phenolic antioxidants' ability to scavenge ABTS^+^ either by electron transfer or by donation of hydrogen atom or a combination of both [30]. Nitric oxide (NO) is a vital chemical produced by neurons, macrophages, and endothelial cells that is associated with the control of different physiological process, but its production in excess concentration has been associated with several human diseases. Oxygen reacts with excess NO to produce nitrite and peryoxynitrite anions which are free radicals [40]. Antioxidant ability of plant extracts has been measured based on their ability to scavenge free radicals produced from NO.

The result from the study showed that the extracts of the* Capsicum* varieties exhibited strong antioxidant activities, particularly the ethanolic extracts. Determination of half inhibitory concentration (IC_50_) was used to measure the antioxidant efficacy of the samples as lower values of IC_50_ indicate great capacity in scavenging free radicals [2]. The antioxidant capacities of plants have also been linked to their phytochemical constituents [3]. The presence of appreciable high amount of phenols, flavonoids, and proanthocyanidins in the samples contributes to their antioxidant activities. It is evident from the results that considerable variation existed in the antioxidant capacities among the varieties of* Capsicum *used in the different assays. It is however interesting to report that the ethanolic extract of* Capsicum frutescens* var.* baccatum* gave the highest antioxidant activity in ABTS, NO, and phosphomolybdenum assays when compared to the three varieties from* Capsicum annuum* species. Aqueous extract of* C*.* annuum* var.* acuminatum* has the best scavenging potential against DPPH radical when compared with other varieties. The differences observed in the antioxidant activities among the varieties could be due to impact of genotype, complexity, and diversity of antioxidant compounds available in them [39].

The result from the multivariate analysis revealed that cluster analysis is a resourceful method for variability and similarity studies in* Capsicum* species. Clustering of genotypes into groups was based on genetic similarity of their bioactive constituents and genotypes that clustered in similar groups possess common genetic affinities which are the basis for taxa delineation. The resulting dendrogram in this study distinguished the species into 2 different clusters where it distinctly separated* Capsicum frutescens* var.* baccatum* from the varieties of* Capsicum annuum*. This supports the proposition that* C*.* frutescens *and* C*.* annuum* are distinct species. However, higher affinity was observed between* C*.* frutescens* var.* baccatum *and* C*.* annuum* var.* abbreviatum* than the other varieties. Similar result from cluster analysis was seen in our previous work on morphological characterization of these species [41]. A rational selection of these genotypes with genetic affinity as parental stock could generate higher improvement in a hybridization programme.

## 5. Conclusion

The evaluation of levels of genetic variation which aids in proper delineation of plant species is fundamental in agriculture as it helps to effectively conserve, manage, and develop improved cultivars of plants that are endowed with bioactive compounds for various pharmacological uses. Determination of the level of variation of plant bioactive compounds has been successfully used as an additional tool for classification in some plant species. In this study, evaluation of phytochemical content and antioxidant activities proved to be useful in the continuous quest to better characterize and classify the varieties and the species of* Capsicum* in West Africa. Dendrogram obtained from the multivariate analysis distinctly separated* C*.* frutescens* var.* baccatum* from the varieties of* C*.* annuum*. This corroborates the proposition that* C*.* annuum* and* C*.* frutescens* are distinct species. The clustering of* C*.* frutescens* var.* baccatum* with* C*.* annuum *var.* abbreviatum* suggests its closeness genetically with this variety compared to the others and affirmed that all varieties share a common progenitor. Additional comparative study using DNA profiling (which is on-going) is needed to better characterize these species. More importantly, the high phytochemical contents and antioxidant activities displayed in the various extracts of the varieties of* Capsicum* species indicate their pharmacological and nutritional value. Consumption of this beneficial crop as a source of natural antioxidant is recommended.

## Figures and Tables

**Table 1:** Percentage yields of the four varieties of cultivated *Capsicum* species after extraction.

Varieties	Ethanolic extract	Aqueous extract
*C. annuum* var. *abbreviatum*	12.15	25.66
*C. annuum* var. *acuminatum*	12.46	30.00
*C. annuum* var. *grossum*	12.15	28.00
*C. frutescens* var. *baccatum*	15.08	24.33

**Table 2:** Total phenol, flavonoid, and proanthocyanidins contents in the varieties of the cultivated *Capsicum* species.

Phytochemicals	Phenol (mg GAE/g DW)		Flavonoid (mg QE/g DW)		Proanthocyanidins (mg CE/g DW)	
Samples	Ethanol	Aqueous	Ethanol	Aqueous	Ethanol	Aqueous

*C. annuum var. abbreviatum*	236.08±7.44^b^	70.11±1.50^b^	1605.36±49.75^a^	373.14±6.40^b^	619.96±6.00^b^	444.95±2.19^a^

*C. annuum var. acuminatum*	200.70±11.53^c^	75.22±2.64^b^	1223.71±100.01^b^	386.70±14.93^b^	629.22±11.16^b^	431.71±8.06^a^

*C. annuum var. grossum*	272.47±7.38^a^	57.36±2.50^c^	1630.53±86.96^a^	317.73±3.69^c^	709.99± 5.50^a^	431.07±13.75^a^

*C. frutescens var. baccatum*	221.21±6.37^b^	90.86±3.78^a^	867.241±53.87^c^	543.09±7.39^a^	616.81±12.34^b^	444.32±12.89^a^

Values are mean ± SD. Samples within a column having different letters are significantly different at (p<0.05).

**Table 3:** IC_50_ values of the varieties of *Capsicum *spp. extracts and standard drugs.

	DPPH		ABTS		Nitric oxide		Phosphomolybdenum	
Sample/standard	Ethanolic	Aqueous	Ethanolic	Aqueous	Ethanolic	Aqueous	Ethanolic	Aqueous

*C. annuum* var. *abbreviatum*	0.0779	0.0523	0.0033	0.0046	0.1803	> 0.4	0.0902	0.2635
*C. annuum* var. *acuminatum*	> 0.08	0.0153	0.0103	0.0047	0.2379	> 0.4	0.1636	0.2171
*C. annuum* var. *grossum*	> 0.08	0.0261	0.0067	0.0043	0.2395	> 0.4	0.0951	0.3522
*C. frutescens* var. *baccatum*	> 0.08	0.0206	0.0024	0.0031	0.0971	> 0.4	0.0892	0.2651
Rutin	0.0059	0.0059	0.0057	0.0057	> 0.4	> 0.4	0.0743	0.0743
Gallic	-	-	0.0046	0.0046	-	-	0.0485	0.0485
BHT	-	-	0.0074	0.0074	0.2075	0.2075	0.374	0.374

## Data Availability

The data supporting the research will be made available by request.
